# Serum Resistin, Cardiovascular Disease and All-Cause Mortality in Patients with Type 2 Diabetes

**DOI:** 10.1371/journal.pone.0064729

**Published:** 2013-06-03

**Authors:** Claudia Menzaghi, Simonetta Bacci, Lucia Salvemini, Christine Mendonca, Giuseppe Palladino, Andrea Fontana, Concetta De Bonis, Antonella Marucci, Elizabeth Goheen, Sabrina Prudente, Eleonora Morini, Stefano Rizza, Alyssa Kanagaki, Grazia Fini, Davide Mangiacotti, Massimo Federici, Salvatore De Cosmo, Fabio Pellegrini, Alessandro Doria, Vincenzo Trischitta

**Affiliations:** 1 Research Unit of Diabetes and Endocrine Diseases, IRCCS Casa Sollievo della Sofferenza, San Giovanni Rotondo, Italy; 2 Unit of Endocrinology, IRCCS Casa Sollievo della Sofferenza, San Giovanni Rotondo, Italy; 3 Research Division, Joslin Diabetes Center, Boston, Massachusetts, United States of America; 4 Unit of Biostatistics, IRCCS Casa Sollievo della Sofferenza, San Giovanni Rotondo, Italy; 5 IRCSS Casa Sollievo della Sofferenza-Mendel Laboratory, Rome, Italy; 6 Department of Internal Medicine, University of Rome Tor Vergata, Rome, Italy; 7 Unit of Biostatistics, Consorzio Mario Negri Sud, Santa Maria Imbaro, Italy; 8 Department of Medicine, Harvard Medical School, Boston, Massachusetts, United States of America; 9 Department of Experimental Medicine, Sapienza University of Rome, Italy; University of Siena, Italy

## Abstract

**Background:**

High serum resistin has been associated with increased risk of cardiovascular disease in the general population, Only sparse and conflicting results, limited to Asian individuals, have been reported, so far, in type 2 diabetes. We studied the role of serum resistin on coronary artery disease, major cardiovascular events and all-cause mortality in type 2 diabetes.

**Methods:**

We tested the association of circulating resistin concentrations with coronary artery disease, major cardiovascular events (cardiovascular death, non-fatal myocardial infarction and non-fatal stroke) and all-cause mortality in 2,313 diabetic patients of European ancestry from two cross-sectional and two prospective studies. In addition, the expression of resistin gene (*RETN*) was measured in blood cells of 68 diabetic patients and correlated with their serum resistin levels.

**Results:**

In a model comprising age, sex, smoking habits, BMI, HbA1c, and insulin, antihypertensive and antidyslipidemic therapies, serum resistin was associated with coronary artery disease in both cross-sectional studies: OR (95%CI) per SD increment = 1.35 (1.10–1.64) and 1.99 (1.55–2.55). Additionally, serum resistin predicted incident major cardiovascular events (HR per SD increment = 1.31; 1.10–1.56) and all-cause mortality (HR per SD increment = 1.16; 1.06–1.26). Adjusting also for fibrinogen levels affected the association with coronary artery disease and incident cardiovascular events, but not that with all cause-mortality. Finally, serum resistin was positively correlated with *RETN* mRNA expression (rho = 0.343).

**Conclusions:**

This is the first study showing that high serum resistin (a likely consequence, at least partly, of increased *RETN* expression) is a risk factor for cardiovascular disease and all-cause mortality in diabetic patients of European ancestry.

## Introduction

Cardiovascular disease (CVD) is a major cause of morbidity and mortality among patients with type 2 diabetes [1]. Although several components of the diabetic milieu contribute to the increased risk of CVD associated with diabetes, insulin resistance and inflammation have been recognized as particularly important pathogenic factors [2]. Both conditions have been linked to cytokines released by the adipose tissue and collectively known as adipokines [3]. Among these is resistin, a 12.5 kDa cysteine-rich protein, which, in humans, is primarily secreted by macrophages [4], [5]. Several cross-sectional studies based on resistin serum levels and/or tissue expression have pointed to this molecule as a pro-inflammatory adipokine contributing to atherosclerosis and the clinical phenotypes resulting from it [5], [6], [7], [8], [9], [10], [11]. High serum resistin levels have also been found, although with some inconsistencies, to predict incident cardiovascular events in prospective studies [12], [13], [14], [15], [16], [17]. This evidence, however, mostly concerns the general population since the few published studies of resistin as a CVD marker in diabetic subjects are small, limited to Asian individuals, and contradictory in their findings [9], [11], [18]. Given that cardiovascular risk may be differently shaped in non diabetic as compared to diabetic individuals answering the question of whether or not resistin plays a role in the development of CVD also among the latter group is definitely needed. To address this question, we analyzed data from over 2,300 European subjects with type 2 from four different studies: two case-control collections of such patients with and without evidence of coronary artery disease (CAD), a prospective cohort of patients with type 2 diabetes followed over time for incident major cardiovascular events and another prospective cohort of patients with type 2 diabetes followed over time with regard to all-cause mortality.

## Methods

### Case-Control Studies

#### Gargano heart study (GHS)-cross sectional design

This study includes 798 European subjects from Italy with type 2 diabetes (ADA 2003 criteria) who were consecutively recruited at the Endocrine Unit of IRCCS “Casa Sollievo della Sofferenza” in San Giovanni Rotondo (Gargano, Center East Coast of Italy) from 2001 to 2008, as part of an ongoing investigation on the genetics of CAD in type 2 diabetes [19], [20] (Figure S1).

Cases are patients who underwent coronary angiography and had a stenosis >50% in at least one coronary major vessel or with previous myocardial infarction (MI). Controls include asymptomatic patients without signs of myocardial ischemia at resting and maximal symptom limited stress ECG. The latter was conducted on a treadmill according to a Bruce protocol after cardiovascular drugs as β-blockers and Ca-channel blockers were stopped for 48 hours. The test was defined as maximal if 85% of the predicted heart rate for the participant’s age was reached. Ischemia was defined as a horizontal or downsloping ST segment depression of 1 mm or more calculated at 0.08 s after the J point (i.e. the junction between QRS complex and ST segment) or the development of typical angina pectoris.

Serum resistin was measured in 776 (97%) participants.

#### Joslin Heart Study (JHS)

This study consists of a series of 868 CAD cases and controls, all with type 2 diabetes (ADA 2003 criteria), who lived in the greater Boston area and received treatment at the Joslin Clinic and/or the Beth Israel Deaconess Medical Center (BIDMC) at the time of their recruitment [21].

All participants were self-reported non-Hispanic Whites. Case participants with CAD were a random sample of patients with type 2 diabetes who had a stenosis greater than 50% in a major coronary artery or a main branch thereof that was documented by cardiac catheterization at the BIDMC between 2001 and 2008. Sixty percent of the case patients received diabetes management care at the Joslin Clinic. Control participants without CAD were randomly selected from among Joslin patients who were identified between 2001 and 2008 as fulfilling the following criteria: (1) current age between 55 and 74 years; (2) type 2 diabetes for 5 years or more; (3) negative cardiovascular history (i.e., normal resting electrocardiogram, absence of cardiac symptoms, and no hospitalization for cardiovascular events); and (4) non inducible ischemia to an exercise treadmill test performed for screening purposes.

Serum resistin was measured in 861 (99%) participants.

### Prospective Studies

#### GHS-prospective design

This study comprises 368 patients with type 2 diabetes and CAD (as previously defined), who were all case participants of the GHS-cross sectional design (Figure S1). Follow-up information on outcomes was collected yearly from 2002 to 2011. The only exclusion criterion was the presence of poor life expectancy for non diabetes-related diseases. The end-point was a combination of major cardiovascular events including cardiovascular death (i.e. according to the international classification of diseases’ codes: 428.1- ninth edition - and I21.0–I21.9, I25.9, I46.9–I50.9, I63.0, I63.9, I70,2– tenth edition), non-fatal MI and non-fatal stroke [22]. For all non-fatal MI and strokes, confirmation of the events was obtained from the hospital medical records. In the case of patients who did not show up at the scheduled clinical control, information on the incident cardiovascular events was obtained through telephone interviews with the patients or their primary care physicians or from death certificates.

Serum resistin was measured in 359 (98%) participants.

#### Gargano Mortality Study (GMS)

One thousand and twenty-eight patients with type 2 diabetes (ADA 2003 criteria) were consecutively recruited from November 1th 2000 to September 30th 2005 at the Endocrine Unit of IRCCS “Casa Sollievo della Sofferenza” in San Giovanni Rotondo, for a study having all-cause mortality as the end-point (Figure S1). The only exclusion criterion was the presence of poor life expectancy due to non diabetes-related disorders. This cohort was followed until 2010 by obtaining information on the participants’ vital status by direct contact with patients and/or their relatives or by queries to the registry offices of the cities of residence. Such information was available in 838 individuals whose data were therefore analyzed in the present study. One hundred and three of the GMS participants are also participants of the GHS-prospective design (Figure S1).

Serum resistin was measured in 779 (93%) participants.

### Data Collection and Definitions

Clinical data were obtained from a standardized interview and examination. Body mass index (BMI) was calculated by dividing the weight (in kilograms) by the square of height (in meters). Smoking habits and history of hypertension (as indicated by the presence of anti-hypertensive therapy), dyslipidemia (as indicated by the presence of anti-dyslipidemic therapy), and MI as well as glucose-lowering treatment were also recorded at time of examination. Data regarding medications were confirmed by review of medical records. Those who reported smoking cigarettes regularly during the year before the examination were considered current smokers.

In the GHS-cross sectional and-prospective designs and GMS, blood samples were collected between 8∶00 and 9∶00 AM after an overnight fast. In the JHS, blood samples were obtained between 7∶00 AM and 6∶00 PM without the requirement of fasting. Serum aliquots were stored at −80°C. Peripheral whole blood cells (PWBC) RNA was obtained from 68 fasting patients with type 2 diabetes, with no clinical evidence of CVD (38 males/30 females, age 65.1±7.0 years, BMI 30.9±5.0 kg/m^2^, HbA1c 7.9±1.7%) by PAXgene Blood RNA collection tubes (PreAnalytiX, GmbH, Germany). These patients, not belonging to any of the previous samples, were consecutively recruited at the Endocrine Unit of IRCCS “Casa Sollievo della Sofferenza” in San Giovanni Rotondo, with the specific purpose of correlating gene expression levels on PWBC with clinical features and/or biomarkers levels.

### Ethics

Each study protocol and the informed consent procedures were approved by the local Institutional Ethic Committee IRCCS (Istituto di Ricovero e Cura a Carattere Scientifico) “Casa Sollievo della Sofferenza” for GHS and GMS and by the Joslin Committee on Human Studies and the Beth Israel Deaconess Medical Center Committee on Clinical Investigations for JHS. All participants gave written consent.

### Measurement of Circulating Resistin Levels

Serum resistin concentrations were measured by a commercial ELISA (Bio Vendor, Brno Czech Republic) at the Research Unit of Diabetes and Endocrine Diseases in San Giovanni Rotondo, as previously described [23]. Inter- and intra-assay coefficients of variation were 3.2–4% and 6.3–7.2% respectively.

### Measurement of *RETN* mRNA Levels

Total RNA from PWBC was extracted using PAXgene Blood RNA kit (PreAnalytiX, GmbH, Germany). RNA was eluted in RNAse free-water and stored at *−*80*°*C until used. Total RNA yield and purity were determined spectrophotometrically using the NanoDrop ND-1000 (Wilmington, DE, USA). Integrity of resuspended total RNA was determined by electrophoretic separation and subsequent laser induced florescence detection using the RNA 6000 Nano Assay Chip Kit on the Bioanalyzer 2100 (Agilent Technologies, Waldbronn, Germany).

Five hundred nanograms of RNA were reverse transcripted by AMV Reverse Transcription System (Promega Corp., Wis, USA) and used as template in subsequent analyses. *RETN* (Hs00220767_m1) and *GAPDH* (Hs99999905_m1) gene expression assays on demand kit reagents Applied Biosystems (Foster City, CA) were used to quantify in triplicates relative gene expression on ABI-PRISM 7500 Applied Biosystems (Foster City, CA). *RETN* transcription levels were normalized using the *GAPDH* housekeeping gene. *RETN*/*GAPDH* mRNA ratios were obtained from the equation 2^−ΔCt^, where ΔCt is the difference in threshold cycles between *RETN* and *GAPDH*.

### Statistical Methods

Patients’ baseline characteristics were reported as mean±standard deviation (SD) and percentages for continuous and categorical variables, respectively. Correlations between continuous variables were assessed by Pearson coefficient.

In case-control studies, the association between resistin circulating levels and CAD was assessed with univariate and multivariate logistic regression models with CAD status as the dependent and resistin as the independent variable. Separate analyses were performed for continuous resistin values and tertiles of its distribution. The strength of the associations was estimated by means of odds ratios (ORs), along with their 95% Confidence Intervals (95% CI), per SD increase in baseline resistin level and for tertiles of its distribution. In addition, a test for linear trend in OR estimates over resistin tertiles was performed by including resistin tertiles (coded as 1, 2, and 3) as a continuous variable into the logistic model.

In both prospective studies, the time variable was defined as the time between the baseline examination and date of the event (namely, major cardiovascular events for GHS-prospective, and all-cause mortality for GMS), or, for subjects who did not experience any event, the date of the last available clinical follow-up. Incidence rates for the endpoint of interest were expressed as the number of new events per total number of person-years (py) and were compared between baseline serum resistin levels tertiles using a Poisson regression model. In addition, a test for linear trend in incidence rates over resistin tertiles was performed by including resistin tertiles as a continuous variable into the Poisson model.

Univariate and multivariate Cox proportional hazards regressions analyses were performed to assess the association between resistin values or tertiles of its distribution and the event occurrence. Risks were reported as Hazard Ratios (HR) along with their 95% CI per SD increase in resistin levels and for tertiles of its distribution. Test for linear trend in HR estimates over resistin tertiles was performed by including resistin tertiles as a continuous variable into the Cox proportional hazard models. Adjusted survival curves were derived from the Cox proportional hazard models, using the direct approach [24].

Predicted risk probabilities were derived from the Framingham Risk Score (FRS), which is an established risk model for cardiovascular event in the general population [25] and from the UKPDS risk engine, which is a model for the risk of coronary heart disease in type 2 diabetes [26]. Models’ calibration, i.e. the agreement between observed outcomes and predictions, was assessed using the survival-based Hosmer-Lemeshow (HL) goodness-of-fit test [27], a chi-square test based on grouping observations into deciles of predicted risk and testing associations with observed outcomes. Models’ discrimination, i.e. the ability to distinguish subjects who will develop an event from those who will not, was assessed by computing the modified C statistic for censored survival data [28], [29]. Comparison between C-indices was carried out following Pencina and D’Agostino’s approach [29].

Reclassification improvement offered by resistin was quantified using the survival-based net reclassification index (NRI) following the Kaplan-Meier approach with one-sided bootstrapped p-values based on 1000 re-samplings with replacement [30], [31] and by Integrated Discrimination Improvement (IDI) (28). Since no established risk cut-offs were available for our high risk patients as those affected by diabetes, we computed the categories-free version of NRI (i.e. cNRI) [31]. The main difference consists in the definition of a reclassified subject: for the NRI a subject has to move from one risk-category to another one; the cNRI requires that the subject’s risk probability changes, without any limit, to define an upward or downward reclassification. cNRI is a more objective measure of improvement in risk prediction while NRI has a more attractive interpretation for clinicians. The time horizon of risk prediction was set to 7 years. (i.e. upper cut-off of the 3th quartile).

A p-value <0.05 was considered as significant. All analyses were performed using SAS Release 9.1.3 (SAS Institute, Cary, NC, USA).

## Results

### Case-Control Studies

**Figure 1:**
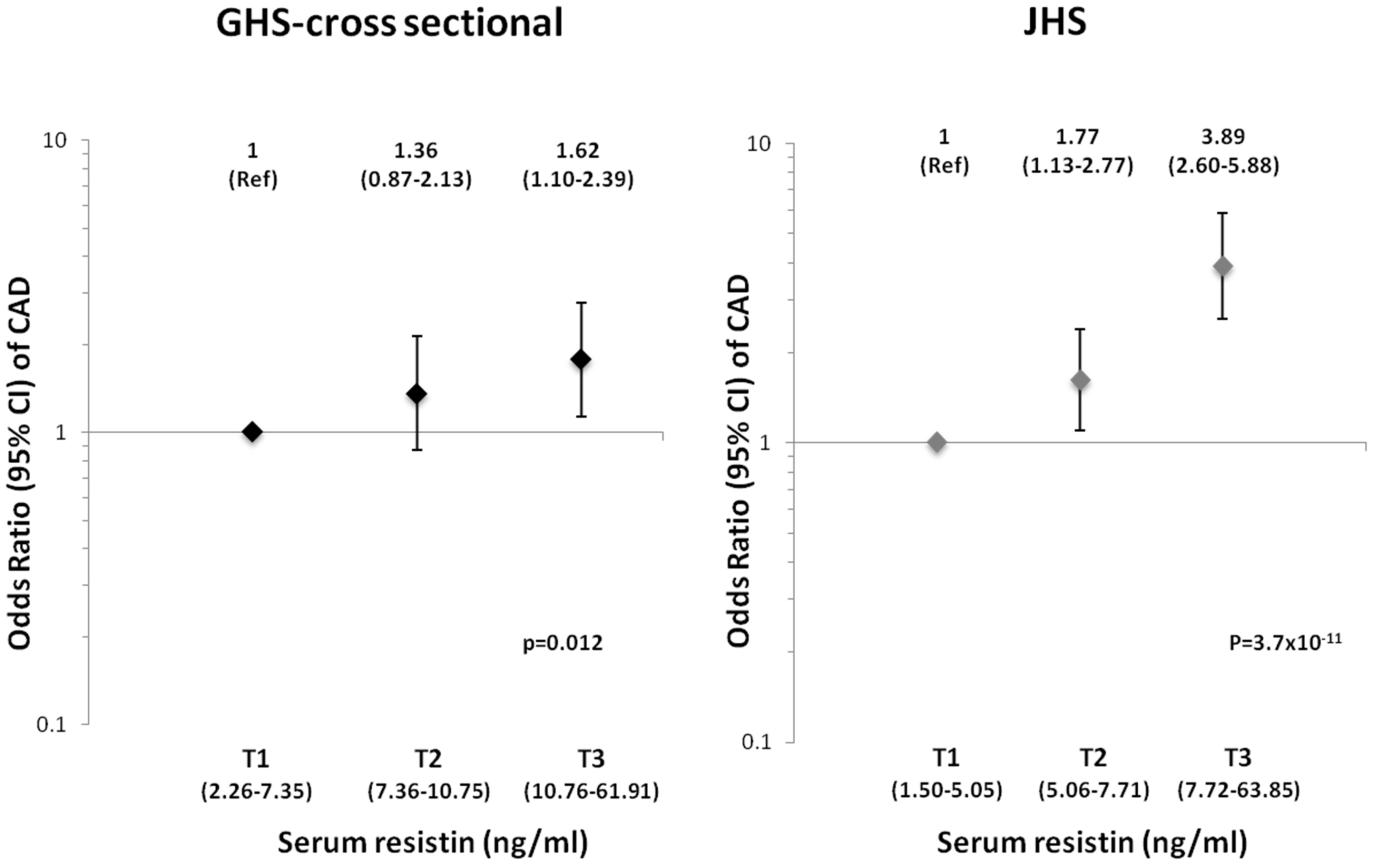
Odds ratios (95% CI) of CAD in cross sectional studies, according to baseline tertile (T1–T3, range in parentheses) of resistin levels. ORs were estimated by logistic regression after adjusting for age, sex, smoking habits, BMI, HbA1c and insulin, antihypertensive and antidyslipidemic therapies.

Clinical features of participants in the GHS-cross sectional design and the JHS are summarized in Table 1. In both studies, serum resistin concentrations were significantly higher in CAD-positive cases than in CAD-negative controls (Table 1). For each SD increment in resistin levels, the odds of CAD increased by ∼30% in the GHS cross-sectional design (OR = 1.29, 95% CI: 1.10–1.51, p = 0.002) and by ∼80% in the JHS (OR = 1.83, 95% CI: 1.49–2.24; p = 3.53×10^−12^) (Table 2). This association was unaffected by adjustments for age, sex, smoking habits, BMI, HbA1c, and insulin, antihypertensive and antidyslipidemic therapies (Table 2). The linear relationship between resistin levels and CAD risk was confirmed in an analysis by resistin tertiles (Figure 1). In both studies, individuals in the second tertile had an OR of CAD that was intermediate between the first and third tertiles, with p-values for linear trend of 0.012 for the GHS-cross sectional design and 3.76×10^−11^ for the JHS (Figure 1).

**Table 1:** Clinical Characteristics of patients from case-control studies.

	GHS-cross sectional design		JHS	
	CAD Negative	CAD Positive	CAD Negative	CAD Positive
	n = 416	n = 360	n = 443	n = 418
Males (%)	184 (44.2)	246 (68.3)	250 (56.4)	304 (72.7)
Age (yrs)	59.9±8.6	64.4±8.1	64.3±6.3	64.3±7.5
Smokers (%)	122 (29.3)	160 (44.4)	169 (38.1)	274 (65.6)
Diabetes duration (yrs)	11.1±8.3	13.8±9.2	12.6±6.7	12.7±8.8
BMI (kg/m^2^)	31.5±5.3	30.2±4.8	32.3±5.6	32.2±6.0
HbA1c (%)	8.5±1.9	8.7±1.9	7.3±1.2	7.4±1.4
Glucose-lowering therapy				
Diet only (%)	41 (9.9)	22 (6.1)	30 (6.8)	24 (5.8)
Oral agents (%)	215 (51.7)	128 (35.6)	225 (51.3)	188 (45.4)
Insulin w/wo oral agents (%)	142 (34.1)	195 (54.2)	184 (41.9)	202 (48.8)
Antihypertensive therapy (%)	279 (67.1)	306 (85.0)	323 (72.9)	365 (87.3)
Antidyslipidemic therapy (%)	141 (33.9)	235 (65.3)	312 (70.4)	343 (82.0)
Fibrinogen (mg/dl)	348.6±87.8	389.3±128.7	N.A.	N.A.
Resistin (ng/ml)	9.33±5.03	10.72±6.68	6.46±4.25	8.64±5.99

Continuous variables were reported as mean±SD whereas categorical variables were reported as total frequency and percentages. GHS: Gargano Heart Study; JHS: Joslin Heart Study; CAD: Coronary Artery Disease; BMI: Body Mass Index; HbA1c: glycated haemoglobin. N.A: Not Available.

**Table 2:** Association between serum resistin levels and CAD occurrence in case-control studies.

	GHS-cross sectional design		JHS	
	CAD Negative n = 416	CAD Positive n = 360	CAD Negative n = 443	CAD Positive n = 418
	OR (95% CI)	p-value	OR (95% CI)	p-value
Model 1	1.29 (1.10–1.51)	0.002	1.83 (1.49–2.24)	3.53×10^−12^
Model 2	1.35 (1.10–1.64)	0.002	1.99 (1.55–2.55)	2.95×10^−13^
Model 3	1.10 (0.93–1.30)	0.260	N.A.	N.A.

GHS: Gargano Heart Study; JHS: Joslin Heart Study; CAD: Coronary Artery Disease.

OR (95% CI) are given for SD increase of resistin levels.

Model 1: unadjusted.

Model 2: adjusted for age, sex, smoking habits, BMI, HbA1c, insulin therapy, hypertension and dyslipidemia.

Model 3: adjusted for plasma fibrinogen.

N.A: Not Available.

Given the role of resistin in low-grade inflammation, we also tested plasma fibrinogen as a covariate. After adjustment for this variable, the association between resistin and CAD in the GHS-cross sectional design was no longer significant (Table 2). Data on fibrinogen levels were not available for the JHS.

It is of note that serum resistin levels in CAD negative controls from both GHS-prospective design and JHS were significantly higher than those previously measured in our laboratory [23] in non diabetic controls (p<0,001 and <0,01, respectively; data not shown).

### Prospective Studies

#### The GHS-prospective design

The clinical features of study participants are summarized in Table 3. During follow-up (5.4±2.5 years), 58 cardiovascular deaths, 6 non-fatal MIs and 9 non-fatal strokes occurred, corresponding to an overall annual incidence rate of 3.8% (73 events/1,934 py). Given that this cohort comprises only very high risk individuals (i.e. diabetic patients who already suffered by coronary stenosis and/or previous MI), it is not surprising that most of major cardiovascular events are represented by death. Each SD increment of serum resistin levels was associated with a 31% increase in the risk of major cardiovascular events (HR = 1.31, 95% CI: 1.13–1.53; p = 3.38×10 ^–4^). As in the case-control studies, the association was not affected by adjustment for age, sex, smoking habits, BMI, HbA1c, and insulin, antihypertensive and antidyslipidemic therapies (HR = 1.31, 95% CI: 1.10–1.56; p = 0.003), but was attenuated and lost significance after adjusting for fibrinogen levels (HR = 1.18, 95% CI: 0.97–1.45; p = 0.099).

**Table 3:** Clinical characteristics of patients from prospective studies.

	GHS-prospective design	GMS
	n = 359	n = 779
Males (%)	242 (67.4)	397 (50.7)
Age (yrs)	64.5±8.1	62.1±9.5
Smokers (%)	160 (44.5)	177 (22.6)
Diabetes duration (yrs)	13.8±9.2	10.9±9.1
BMI (kg/m^2^)	30.2±4.8	31.0±5.6
HbA_1C_(%)	8.6±1.9	8.7±1.9
Glucose-lowering therapy		
Diet only (%)	23 (6.6)	107 (13.7)
Oral agents (%)	127 (35.4)	326 (41.6)
Insulin w/wo oral agents (%)	194 (54.0)	326 (41.6)
Antihypertensive therapy (%)	305 (85.0)	405 (51.7)
Antidyslipidemic therapy (%)	233 (64.9)	261 (33.3)
Fibrinogen (mg/dl)	385.9±124.1	365.3±109.6
Resistin (ng/ml)	10.7±6.6	10.1±8.1

Continuous variables were reported as mean±SD whereas categorical variables as total frequency and percentages. GHS: Gargano Heart Study; GMS: Gargano Mortality Study; BMI: body mass index; HbA1c: glycated haemoglobin.

**Figure 2:**
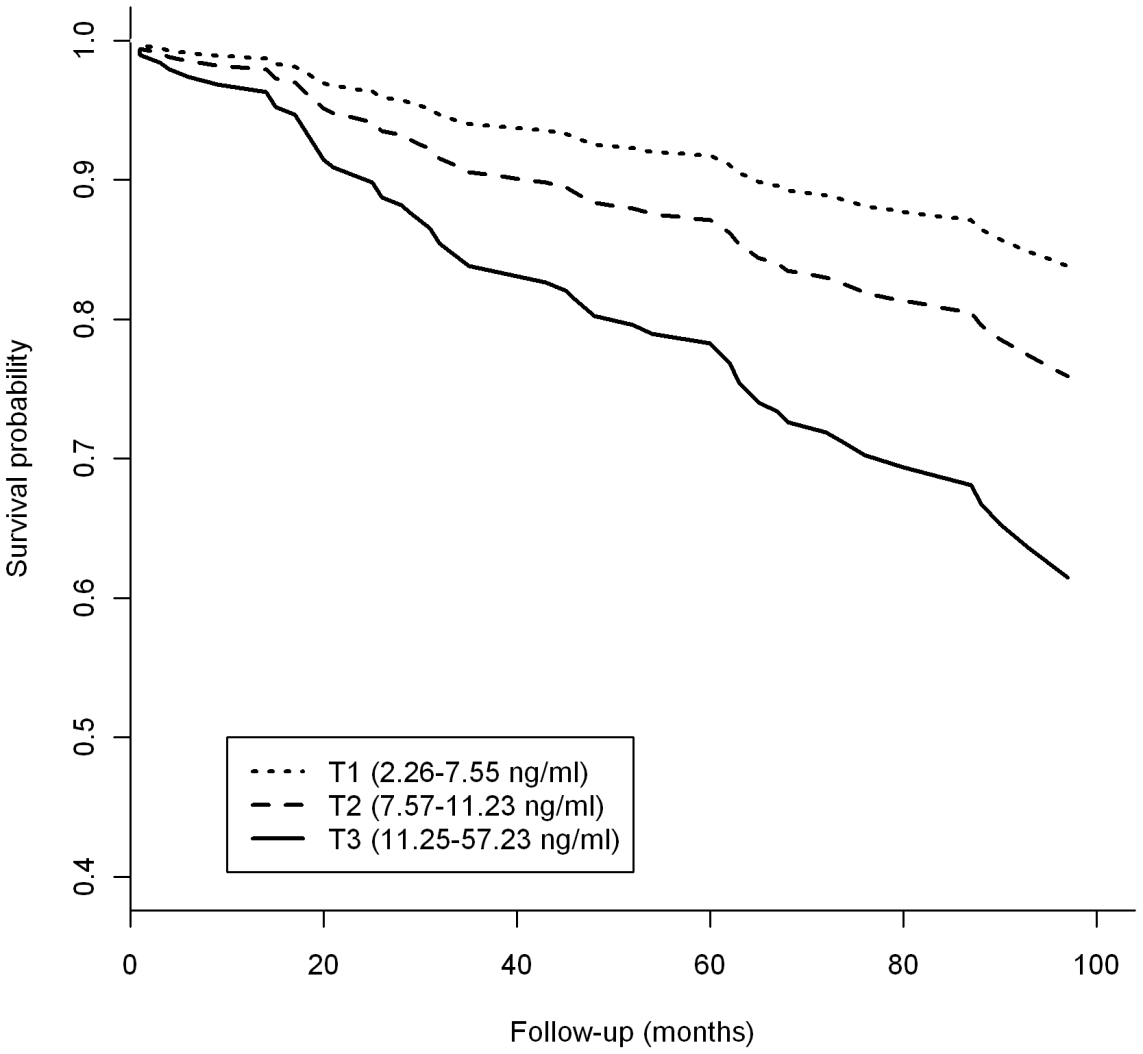
Survival curves for major cardiovascular events in the GHS-prospective design, according to baseline tertile (T1–T3, range in parentheses) of resistin levels. Curves are estimated by Cox regression after adjusting for age, sex, smoking habits, BMI, HbA1c and insulin, antihypertensive and antidyslipidemic therapies.

After stratification by tertiles of baseline resistin levels, the incidence rate of major cardiovascular events was 2.4% (17 events/711 py) in the first, 3.5% (23 events/665py) in the second, and 5.9% (33 events/558 py) in the third tertile (p for trend = 0.001). Accordingly, the HR of major cardiovascular events progressively increased across resistin tertiles, and persisted after adjustment for age, sex, smoking habits, BMI, HbA1c and insulin, antihypertensive and antidyslipidemic therapies (HR = 1.68, 95% CI: 0.80 to 3.53 and HR = 2.79, 95% CI: 1.37 to 5.69 in the second and third tertile, respectively; p for trend = 0.004) (Figure 2).

Survival C statistic, IDI and cNRI indices were used to evaluate the incremental prognostic information of serum resistin for major cardiovascular events as obtained by the FRS [25] and the UKPDS risk engine [26]. Time horizon prediction was set to 7 years, replacing the baseline survival probability accordingly. Patients whose information of some clinical variable used in FRS and/or UKPDS risk engine was not available (n = 61) were excluded.

FRS did not perform well in our sample with survival C statistic being equal to 0.584 (95% CI: 0.510–0.657). The addition of serum resistin produced a significant (p = 0.028) improvement, with survival C statistic becoming 0.640 (95% CI: 0.568–0.713). Both models resulted well calibrated (HL p-values being 0.532 and 0.256, respectively). Moreover, a significant improvement in discrimination was also detected by IDI: 0.022, 95% CI: 0.004–0.048, p = 0.003. Finally, the addition of serum resistin to the FRS allowed to reclassify correctly 95/298 patients (cNRI = 0.433, p = 0.006): 92/244 (37.7%) and 3/54 (5.6%) in those without and with incident events, respectively.

The UKPDS risk engine too performed poorly (survival C statistic = 0.674; 95% CI: 0.607–0.741). The addition of serum resistin produced a significant (p = 0.025) improvement (survival C statistic = 0.704; 95% CI: 0.641–0.766). Both models resulted well calibrated (HL p-values being 0.169 and 0.176, respectively). In contrast, resistin addition did not result in a significant IDI (0.005; 95% CI: −0.01–0.024, p = 0.286). Finally, the addition of serum resistin to the UKPDS risk engine allowed to reclassify correctly 98/298 patients (cNRI = 0.459, p = 0.004): 94/244 (38.5%) and 4/54 (7.4%) in those without and with incident events, respectively.

#### The GMS

Given that CVD is the main cause of death among patients with type 2 diabetes, we assessed the role of serum resistin in predicting all-cause mortality in a cohort of such patients. The clinical features of the GMS participants are summarized in Table 3. During follow-up (7.6±2.1 years), 150 deaths occurred, corresponding to an annual incidence rate of 2.7% (150 events/5,659 py).

Serum resistin predicted the risk for all-cause death with an HR of 1.18 (95% CI: 1.12–1.27; p = 1.5×10^−6^) per SD increment. One hundred and three GMS participants overlapped with those of GHS-prospective design. Exclusion of these subjects did not substantially alter the results (HR = 1.17, 95% CI: 1.07–1.27; p = 0.00047). Similar results were obtained in the whole cohort after adjusting for age, sex, smoking habits, BMI, HbA1c and insulin, antihypertensive and antidyslipidemic therapies (HR = 1.16, 95% CI: 1.06–1.26; p = 0.001).

**Figure 3:**
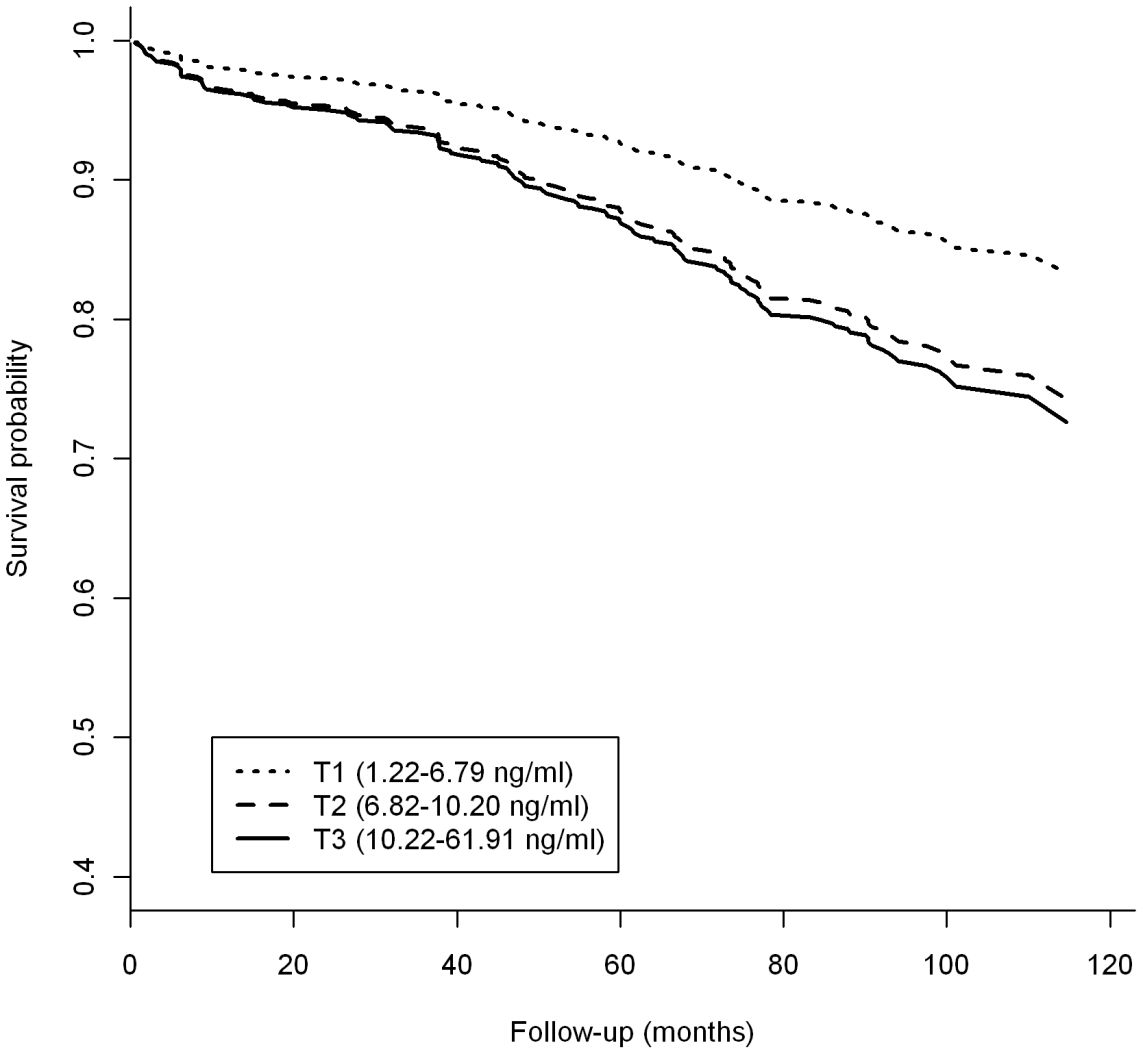
Survival curves for all-cause mortality in the GMS, according to baseline tertile (T1–T3, range in parentheses) of resistin levels. Curves are estimated by Cox regression after adjusting for age, sex, smoking habits, BMI, HbA1c and insulin, antihypertensive and antidyslipidemic therapies.

The all-cause mortality rates in the first, second, and third tertile of resistin levels were 1.8% (36 events/2,022 py), 2.8% (52 events/1,862 py), and 3.5% (62 events/1,775 py), respectively (p for trend = 0.001). At variance with the linear trend observed in both cross-sectional studies and the GHS-prospective design, the adjusted HRs for all-cause mortality were similar in the second and third tertile (HRs = 1.72, 95% CI: 1.11 to 2.68, and 1.81, 95% CI: 1.16 to 2.82, respectively, p for trend = 0.019) (Figure 3). Also at odds with the other studies, the association between resistin and mortality remained significant, though less robustly, after adjusting for fibrinogen levels (HR per resistin SD = 1.15, 95% CI: 1.05–1.27; p = 0.03).

### Correlation between Serum and mRNA Resistin Levels

Since the data above indicate that serum resistin is a marker of cardiovascular risk in patients with type 2 diabetes, we measured *RETN* mRNA levels in circulating PWBC and serum resistin levels in 68 diabetic patients in order to obtain mechanistic insights on the biology of serum resistin variability. The two variables were positively correlated (rho = 0.343, p = 0.006) (Figure S2).

## Discussion

Despite improvements in the treatment of CVD and in the control of risk factors, individuals with type 2 diabetes remain at increased cardiovascular risk as compared to the general population [32] with cardiovascular events being the most important cause of death in these patients [1]. Discovering novel biomarkers able to predict CVD in diabetic patients is therefore urgently needed to decrease the burden of this devastating complication.

To the best of our knowledge, this is the first study to show that elevated serum resistin concentration is a risk factor for CVD in patients with type 2 diabetes of European ancestry. While a rich literature exists on serum resistin as a cardiovascular risk factor in the general population [13], [14], [15], [16], [17], data concerning the type 2 diabetes population have been thus far sparse, contradictory, and limited to Asian individuals [9], [11], [18]. Two small cross-sectional studies of patients with type 2 diabetes from Japan and Korea described an association of serum resistin with CAD and stroke, respectively, but a third study on 343 diabetic Korean patients failed to confirm such findings in a prospective setting. By contrast, we have obtained strong and consistent evidence of association between serum resistin and CVD from both cross-sectional and prospective studies on patients with type 2 diabetes of European ancestry. This effect of resistin is independent of the most established cardiovascular risk factor including sex, smoking habits, BMI, HbA1c and insulin, antihypertensive and antidyslipidemic therapies. However, if the analysis is adjusted for fibrinogen, the association is no longer significant, suggesting it is mediated at least in part by low-grade inflammation – an established cardiovascular risk factor. Whether resistin is a true risk factor which may causally contribute to CVD or, in contrast, a simple biomarker of pro-inflammatory status, cannot be addressed by our study.

We wanted to investigate whether serum resistin provides incremental information in predicting major cardiovascular events as obtained by well established models such as the FRS and the UKPDS risk engine [25], [26] which, similarly to other models, are known not to perform well in the subset of patients with type 2 diabetes [33], [34]. Thus, it was not unexpected that both models performed poorly also in our sample in predicting major cardiovascular events. Of note, the addition of serum resistin improved the two models in terms of both discriminatory and reclassification performance. Such improvement was not only statistically significant, but also of clinical relevance. Further larger studies, are needed to deeper address the relative importance of resistin as an additional marker of clinical utility. for predicting CVD in patients with type 2 diabetes.

Our study also shows for the first time that serum resistin is an independent predictor of all-cause mortality in a study comprising 779 patients with type 2 diabetes. In contrast to what was observed with CVD risk, the association with mortality is only modestly affected by adjustment for fibrinogen levels. Therefore, though fibrinogen is not the best marker of low-grade inflammation, it may be hypothesized that the effects of resistin on all-cause mortality are mediated by mechanisms that are independent of this pathway. The possibility that different mechanisms underlie the effects of resistin on CVD and all-cause mortality is also suggested by the fact that the relationship between resistin levels and all-cause mortality does not appear to be linear as that between resistin and CAD or major CVD events. Consistent with such hypothesis, the association between resistin and all-cause mortality observed in the general population [35], [36], [37] seems to be independent from cardiovascular mortality [35], [36]. Unfortunately, data on cause of death that could confirm this finding among type 2 diabetes patients were not available in the GMS.

An additional finding of our study is that *RETN* mRNA in PWBC is correlated to serum resistin. This result, which is consistent with the observation that human resistin is mainly produced by macrophages [5], strongly suggest that resistin circulating levels are modulated by gene expression levels. The mechanism(s) underlying such modulation are not yet known and need further studies to be unraveled.

One strength of our study is the overall sample size, consisting of a total of 2,313 diabetic patients from two cross-sectional and two prospective investigations, and the completeness of clinical information, including standardized clinical evaluations and hard end-points validated by medical records or death certificates. Another strength is the fact that the resistin measurements were centralized and all the samples were handled identically. In this context, the observed difference in serum resistin concentration between the two cross-sectional studies, with JHS participants having 20–30% lower mean levels as compared to GHS individuals, is somewhat surprising. One possibility is that such difference was due to the different proportion of patients treated with lipid lowering agents in the two studies (77% in the JHS as compared to 48% in the GHS). Such agents are mainly statins, which are known to decrease serum resistin levels [38]. This hypothesis is supported by the observation that participants in the GHS-cross sectional design who were on statins had serum resistin levels 10–15% lower than patients who were not on statins (data not shown). At variance, given that no substantial effects of fasting on resistin levels have been described [39], we can exclude that the observed difference in serum resistin levels between GHS and JHS is due to the different fasting status of the two studies.

Despite these differences, the fact that the association between serum resistin and CAD, that was found in the GHS-cross sectional was fully replicated in the JHS makes our finding especially convincing.

Of note, resistin levels in CAD-negative controls from both GHS-cross sectional and JHS were clearly higher than those from non diabetic controls [23].

The major limitation of our study is represented by the lack of C-reactive protein measurements which surely would have help clarify the link between resistin and chronic inflammation state. In fact, the role of fibrinogen on inflammation remains largely speculative, thus making not possible to draw firm conclusions about the biology underlying the association we observed in our present study.

Finally, whether our finding can be generalized to other populations of different ethnicity having different environmental and/or genetic background remains to be established. This issue deserves particular attention given that a different genetic regulation in different ethnic groups has been hypothesized for serum resistin [40]. Therefore, additional studies are certainly needed to confirm our present finding in a broader context.

In conclusion, our study is the first to show that high serum resistin (a likely consequence, at least in part, of increased resistin mRNA expression) is a risk factor for CVD and all-cause mortality in patients with type 2 diabetes of European ancestry. Further studies are warranted to determine whether this biomarker can be used in a clinical setting to improve the stratification of diabetic patients with regard to their risk for CVD and death.

## Supporting Information

Figure S1 Diagrams and participants of Gargano Heart Study and Gargano Mortality Study in whom serum resistin levels were available. *Gargano Heart Study (GHS)-cross sectional design* includes 776 European subjects with type 2 diabetes mellitus (T2DM), 360 CAD positive and 416 CAD negative as defined in methods. *GHS-prospective design* comprises 359 patients with T2D and CAD who were all case participants of the GHS-cross sectional design as described in methods. *Gargano Mortality Study (GMS)* comprises 779 patients with T2D as describes in methods. One hundred and three of the GMS participants are also participants of the GHS-prospective design (gray box). Raw data on resistin levels and association with related variables can be provided upon request for collaborative purposes.
(TIF)

Figure S2 Correlation between serum and mRNA resistin levels. Correlation between serum and mRNA resistin levels in 68 patients with type 2 diabetes. *RETN* mRNA levels in PWBC are expressed as arbitrary units (AU) of *RETN*/*GAPDH* ratios.
(TIF)
